# Transcriptome Analysis of the Tadpole Shrimp (*Triops longicaudatus*) by Illumina Paired-End Sequencing: Assembly, Annotation, and Marker Discovery

**DOI:** 10.3390/genes7120114

**Published:** 2016-12-02

**Authors:** Jiyeon Seong, Se Won Kang, Bharat Bhusan Patnaik, So Young Park, Hee Ju Hwang, Jong Min Chung, Dae Kwon Song, Mi Young Noh, Seung-Hwan Park, Gwang Joo Jeon, Hong Sik Kong, Soonok Kim, Ui Wook Hwang, Hong Seog Park, Yeon Soo Han, Yong Seok Lee

**Affiliations:** 1 Genomic Informatics Center, Hankyong National University, 327 Chungang-no, Anseong-si, Gyeonggi-do 17579, Korea; s-jiyeon@hanmail.net (J.S.); jeon5894@gmail.com (G.J.J.); kebinkhs@empal.com (H.S.K.); 2 Department of Life Science and Biotechnology, College of Natural Sciences, Soonchunhyang University, 22 Soonchunhyangro, Shinchang-myeon, Asan, Chungchungnam-do 31538, Korea; bioksw@naver.com (S.W.K.); drbharatbhusan4@gmail.com (B.B.P.); hwamux@naver.com (H.J.H.); jong6922@daum.net (J.M.C.); elegangce@naver.com (D.K.S.); 3 Trident School of Biotech Sciences, Trident Academy of Creative Technology (TACT), Chandaka Industrial Estate, Chandrasekharpur, Bhubaneswar, Odisha 751024, India; 4 Biodiversity Conservation & Change Research Division, Nakdonggang National Institute of Biological Resources, 137 Donam 2-gil, Sangju, Gyeongsangbuk-do 37242, Korea; cindysory@naver.com; 5 Department of Applied Biology, Chonnam National University, 77 Yongbong-ro, Buk-gu, Gwangju 61186, Korea; annemi@chonnam.ac.kr; 6 Biological Resource Center, Korea Research Institute of Bioscience and Biotechnology (KRIBB), 181 Ipsin-gil, Jeongeup-si, Jeollabuk-do 56212, Korea; biopark@kribb.re.kr; 7 National Institute of Biological Resources, 42, Hwangyeong-ro, Seo-gu, Incheon 22689, Korea; sokim90@korea.kr; 8 Department of Biology Education, Kyungpook National University, 80 Daehakro, Bukgu, Daegu 41566, Korea; uwhwang@knu.ac.kr; 9 Research Institute, GnC BIO Co., LTD., 36 Banseokro, Yuseong-gu, Daejeon 34069, Korea; 5022daniel@gmail.com; 10 College of Agriculture and Life Science, Chonnam National University, 77 Yongbong-ro, Buk-gu, Gwangju 61186, Korea; hanys@chonnam.ac.kr

**Keywords:** *Triops longicaudatus*, tadpole shrimp, transcriptome, Illumina sequencing, SSRs (simple sequence repeats)

## Abstract

The tadpole shrimp (*Triops longicaudatus)* is an aquatic crustacean that helps control pest populations. It inhabits freshwater ponds and pools and has been described as a living fossil. *T. longicaudatus* was officially declared an endangered species South Korea in 2005; however, through subsequent protection and conservation management, it was removed from the endangered species list in 2012. The limited number of available genetic resources on *T. longicaudatus* makes it difficult to obtain valuable genetic information for marker-aided selection programs. In this study, whole-transcriptome sequencing of *T. longicaudatus* generated 39.74 GB of clean data and a total of 269,822 contigs using the Illumina HiSeq 2500 platform. After clustering, a total of 208,813 unigenes with an N_50_ length of 1089 bp were generated. A total of 95,105 unigenes were successfully annotated against Protostome (PANM), Unigene, Eukaryotic Orthologous Groups (KOG), Gene Ontology (GO) and Kyoto Encyclopedia of Genes and Genomes (KEGG) databases using BLASTX with a cut-off of 1E−5. A total of 57,731 unigenes were assigned to GO terms, and 7247 unigenes were mapped to 129 KEGG pathways. Furthermore, 1595 simple sequence repeats (SSRs) were detected from the unigenes with 1387 potential SSR markers. This is the first report of high-throughput transcriptome analysis of *T. longicaudatus*, and it provides valuable insights for genetic research and molecular-assisted breeding of this important species.

## 1. Introduction

The tadpole shrimp, *Triops* spp. (order: Notostraca; class: Branchiopoda) is a crustacean that inhabits freshwater, ephemeral ponds in arid regions worldwide [1], and it has been described as a living fossil from the late Cretaceous period similar to other members of this ancient crustacean order. This is allegedly due to their virtually unchanged morphology during an evolutionary time scale spanning more than 70 million years [2,3]. This includes the ability to control the size of mosquito populations by consuming *Culex* larvae [4,5], and its utilization as a biological agent to control weeds in paddy fields [6]. The diversification of cryptic species within the genus occurred more recently than this, based on the subtle differences in genetic composition and morphology [7,8,9]. *Triops longicaudatus* is the most widespread notostracan crustacean, being found in North America, South America, the Caribbean, Saudi Arabia, Japan, and the Pacific Islands [10,11,12,13,14,15,16]. There are a number of reports on its distribution morphology, and reproduction [2,10,11,15,17]. In South Korea, the species has been reported since 1986, where it was collected from paddy fields in the cities of Changnyeong and Samcheonpo (Gyeongsangnam-Do Province) [13]. It was registered as an endangered species in South Korea by the Ministry of Environment in 2004. Since then, populations of *T. longicaudatus* have increased through regional conservation measures and it was removed from the endangered species list in 2012. *T. longicaudatus* is economically important species to be used for environmental friendly agriculture. It is proposed that genetic studies involving genome, transcriptom, and gene function analysis will be necessary to preserve the genotypes of this species by assisting in determining their developmental and regulatory functions. Furthermore, the elucidation of cDNA simple sequence repeat (SSR) markers in the putative coding transcripts will be necessary to assess population genetic structure and diversity.

Among the limited number of genomic resources on *T. longicaudatus*, only the mitochondrial DNA sequence is known [18]. The variation in mitochondrial genes has been successfully utilized to identify cryptic lineages of the genus *Triops* [9]. Despite these studies, genetic and genomic information on the species is limited due to the lack of whole genome sequencing, RNA sequencing, expression profiles of transcripts, and microsatellite markers. The traditional method of expressed sequence tag (EST) construction using Sanger sequencing is time consuming and inefficient, producing at best 10,000 sequences. This is likely an insufficient representation of the size of the genome and thus a major limitation of functional research applications [19]. High-throughput next-generation sequencing (NGS) technologies, such as 454 (Roche), Solexa/Illumina (Illumina), and SOLiD (ABI), collect massive amounts of sequencing data in a single run with increased efficiency at an affordable level [20,21]. This technology has enabled genome and transcriptome-level computational analyses [22], leading to the discovery of molecular markers such as SSRs, single nucleotide polymorphisms (SNPs), and quantitative trait loci (QTL) [23]. Because genome sequences are currently unavailable or unreliable in many non-model species, transcriptome sequencing provides direct relevance to the genetic level by measuring the expression of relevant traits [24,25,26]. Among the NGS technologies, Illumina sequencing is a preferred choice due to the generation of short-read sequences with greater coverage [27,28,29,30].

Over the last four to five years, significant progress has been made in characterizing the transcriptome of economically important crustacean species such as *Litopenaeus vannamei*, *Fenneropenaeus chinensis*, *Eriocheir sinensis*, *Macrobrachium nippoense*, *Portunus trituberculatus*, and *Carcinus maenas*. These analyses have provided insights into species biology, the functional regulation of defense signaling pathways, growth and reproduction, and strategies to improve culture productivity. In this study, we present the first massive sequencing data for the tadpole shrimp, *T. longicaudatus*, using the Illumina HiSeq 2500 NGS platform. The assembled and annotated sequencing data were utilized for the large-scale identification of putative functional transcripts. Furthermore, the identification and analysis of SSR loci and SSR markers in the transcriptome will be useful for population genomics and variability studies, further assisting in the marker assisted selection breeding of *T. longicaudatus*.

## 2. Materials and Methods

### 2.1. Ethics Statement

The experiments in this study were performed in accordance with relevant national and international guidelines. Because *T. longicaudatus* is not an endangered or protected species, in Korea, sample collection did not require special permits. Our project was approved by the National Institute of Biological Resources (NIBR), Korea.

### 2.2. Sample Collection and RNA Isolation

Ten individual *T. longicaudatus* were collected from the Metropolitan City (Palgongsan), Gooam, Dong-gu, Daego-si, South Korea, on 10 June 2014.

The adult whole-body tissues of hermaphrodite *T. longicaudatus* (*n* = 10) were pooled and total RNAs were extracted using Trizol reagent (Invitrogen, Carlsbad, CA, USA) and extracted in accordance with the manufacturer’s protocol. The extracted RNA was treated with RNase-free DNase I (Qiagen) to remove the genomic DNA. RNA purity and concentration were measured using a Nanodrop-2000 spectrophotometer (Thermo Scientific, Wilmington, DE, USA). The Bioanalyzer 2100 (Agilent Technologies, Santa Clara, CA, USA) measures RNA quantity and agarose gel electrophoresis. Total RNAs were pooled, purified to obtain mRNA using oligo (dT) magnetic beads, and subsequently fragmented using an RNA Fragmentation Kit (Ambion, Austin, TX, USA).

### 2.3. cDNA Synthesis and HiSeq 2500 Sequencing

First-strand cDNA synthesis was performed using reverse-transcriptase (Invitrogen) and random hexamer-primers. Second-strand cDNA was synthesized using RNase H (Invitrogen) and DNA polymerase I (New England BioLabs, Ipswich, MA, USA). The double-stranded cDNA was end-repaired using T4 DNA polymerase, the Klenow fragment (New England BioLabs), and T4 polynucleotide kinase (New England BioLabs). The end-repaired cDNA fragments were ligated to the PE (paired-end) Adapter Oligo Mix with T4 DNA ligase (New England BioLabs) at room temperature for 15 min. The ligated products were purified and separated by size on a 2% agarose gel. DNA fragments of the desired size (200 ± 25 bp) were excised and sequenced on the Illumina HiSeq 2500 sequencing platform with 2 × 126 bp after validation.

### 2.4. De Novo Assembly and Assessment of De Novo Assemblies

Before de novo transcriptome assembly, the raw reads were cleaned by removing adaptor-only reads (nt length of the recognized adaptor ≤13 and the remaining adaptor-excluded nt length of ≤35), repeated reads, and low-quality reads (Phred quality score ≤20) using Sickle (http://github.com/najoshi/sickle) [31] and Cutadapt (http://cutadapt.readthedocs.io/en/stable/index.html) [32]. High-quality reads were assembled using Trinity software (software version 2013-02-25) with default parameters (100 GB of memory, path reinforcement distance of 50, and minimum allowed length of 200 bp) [33]. The Trinity program assembles reads of a certain length that overlap to form longer fragments without gaps; these are called contigs. The total number of contigs, as well as the mean length, the N_50_ length, and GC% were recorded. The contigs were further assembled into sequences that could not be extended at either end; these are called unigenes (having 94% identity, 30 bp overlap) [34,35]. Such unigenes were subjected to annotation analysis against public protein and nucleotide databases. The assessment of the assembly and annotation completeness we applied the software tool BUSCO (software version 1.1b) [36].

### 2.5. Transcriptome Annotation

For functional annotation, all transcripts were handled as queries and annotated against subject sequences in the Protostome database (PANM-DB) [37], as well as the Unigene and Eukaryotic Orthologous Groups (KOG) databases using the BLASTX and BLASTN programs, with a significant threshold *E*-value of <0.00001 [38]. Gene Ontology (GO) (http://www.geneontology.org) terms were analyzed using BLAST2GO (http://www.blast2go.org/) software, professional version [39]. Subsequently, the GO functional classification of the annotated transcripts was performed using WEGO software (http://wego.genomics.org.cn/cgi-bin/wego/index.pl) [40]. Kyoto Encyclopedia of Genes and Genomes (KEGG) (http://www.genome.jp/kegg/) metabolic pathway analysis was determined by searching against the KEGG database with an *E*-value cutoff of 1E−5 [41].

### 2.6. Identification of SSRs

SSRs in the unigenes (length > 1000 bp) of *T. longicaudatus* were identified using the Perl script program MicroSAtellite (MISA) (http://pgrc.ipk-gatersleben.de/misa/). Search criteria included the number of repetitions for mono-, di-, tri-, tetra-, penta-, and hexa-nucleotides. The minimum repeats were as follows: six for dinucleotides, five for trinucleotides, and four for tetra-, penta-, and hexa-nucleotides. Primers for each SSR were designed using the BatchPrimer3 (http://wheat.pw.usda.gov/demos/BatchPrimer3/) program.

## 3. Results

### 3.1. Illumina Reads and Sequence Assembly

Transcriptome information for the *T. longicaudatus* was characterized from adult whole-body. The Illumina HiSeq 2500 platform generated a total of 323,319,608 paired-end reads (40,738,270,608 bases) were generated with a read length of 100 bp. All raw sequencing data were deposited into the NCBI Sequence Read Archive (SRA) under accession number SRR3961747. After adaptor trimming, a total of 318,610,596 clean sequencing reads (98.54%) were filtered, which were used for further analysis. The mean length, the N_50_ length, and GC% of the obtained clean reads were 124.7 bp, 126 bp, and 48.39%, respectively.

**Figure 1:**
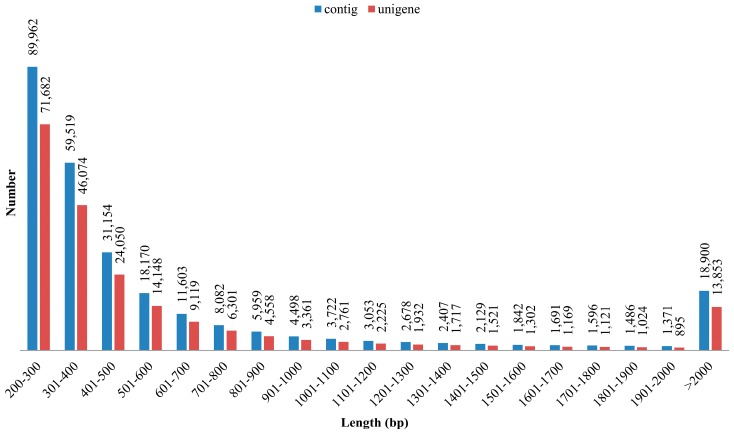
Size distribution of contigs (blue) and unigenes (red) after assembly and clustering of the quality reads from the transcriptome of *T. longicaudatus*.

Because the reference genome sequence is unavailable, de novo assembly of the transcriptome was performed. Trinity assembly with default parameters was used to resolve the clean transcripts to overlapping contiguous sequences. De novo assembly of the high-quality sequences generated a total of 269,822 contigs (192,327,026) with a mean length of 712.8 bp and an N_50_ length of 1148 bp. Of the total assembled contigs, 89,407 were ≥500 bp, with the longest contig size of 40,450 bp. The clustering of the contigs generated 208,813 unigenes with a mean length of 700 bp and an N_50_ length of 1089 bp. The lengths of the unigenes varied from 224 bp to 40,450 bp. Table 1 summarizes the transcriptome sequencing, de novo assembly, and clustering of contigs. Among the unigenes, 85.86%, 7.50%, and 6.63% showed lengths of 200–1000 bp, 1001–2000 bp, and >2000 bp, respectively. The size distribution of the contigs and unigenes are shown in Figure 1. The unigenes represent a comprehensive resource of functional information on the *T. longicaudatus* genome and may facilitate the discovery of relevant phenotypes in this species.

### 3.2. Sequence Annotation of Unigenes

**Figure 2:**
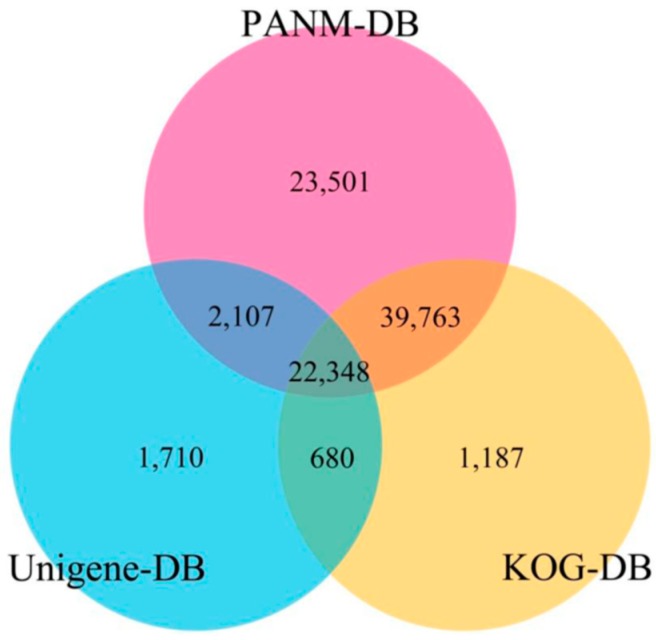
The sequence annotation profile of *T. longicaudatus* unigenes against PANM-DB, Unigene DB and KOG DB.

Several public databases comprised of known protein and nucleotide sequences were used as subject databases for the sequence annotation of *T. longicaudatus* unigenes. The unigene sequences (as queries) were searched to identify homologous sequences using BLASTX and BLASTN (*E*-value cut-off of 1E−5) for protein and nucleotide databases, respectively. The PANM-DB, KOG, GO, and KEGG databases were used as protein databases, while the Unigene database was used as the nucleotide database. Of the total of 208,813 unigenes, 95,105 (45.55%) were annotated to any one of the databases with a great number of unigenes having lengths of 300–1000 bp. The number of matches to PANM-DB was the greatest (87,719 unigenes), followed by the KOG (63,978 unigenes). The annotation results of unigenes to the public databases are shown in Table 2. The results also show that 23,732 (27.1%), 7729 (28.8%), 20,131 (31.55%), 16,663 (28.8%), and 2112 (29.1%) of the unigenes that were over 1000 bp in length had BLAST matches in the PANM, Unigene, KOG, GO, and KEGG databases, respectively. Next, to understand the overlap of the unigene sequence annotations between PANM-DB and Unigene and KOG databases, we constructed a three-way Venn diagram (Figure 2). We found that a maximum number of 39,763 unigenes matched in both PANM-DB and KOG database, and 22,348 unigenes matched in all three databases. The number of unigenes annotated exclusively to PANM-DB, and the Unigene and KOG databases without any overlap were 23,501, 1710, and 1187, respectively.

**Figure 3:**
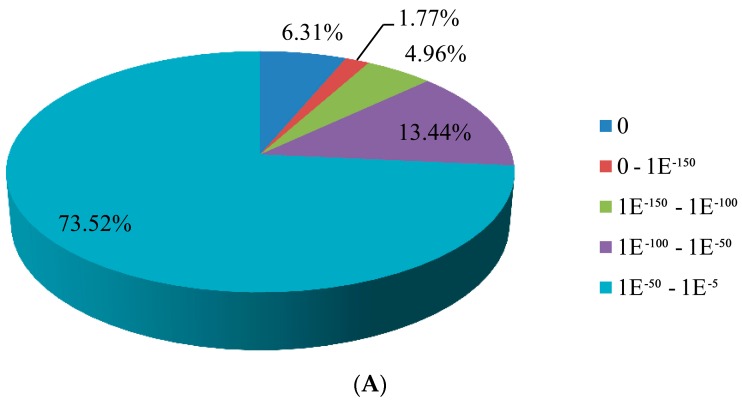

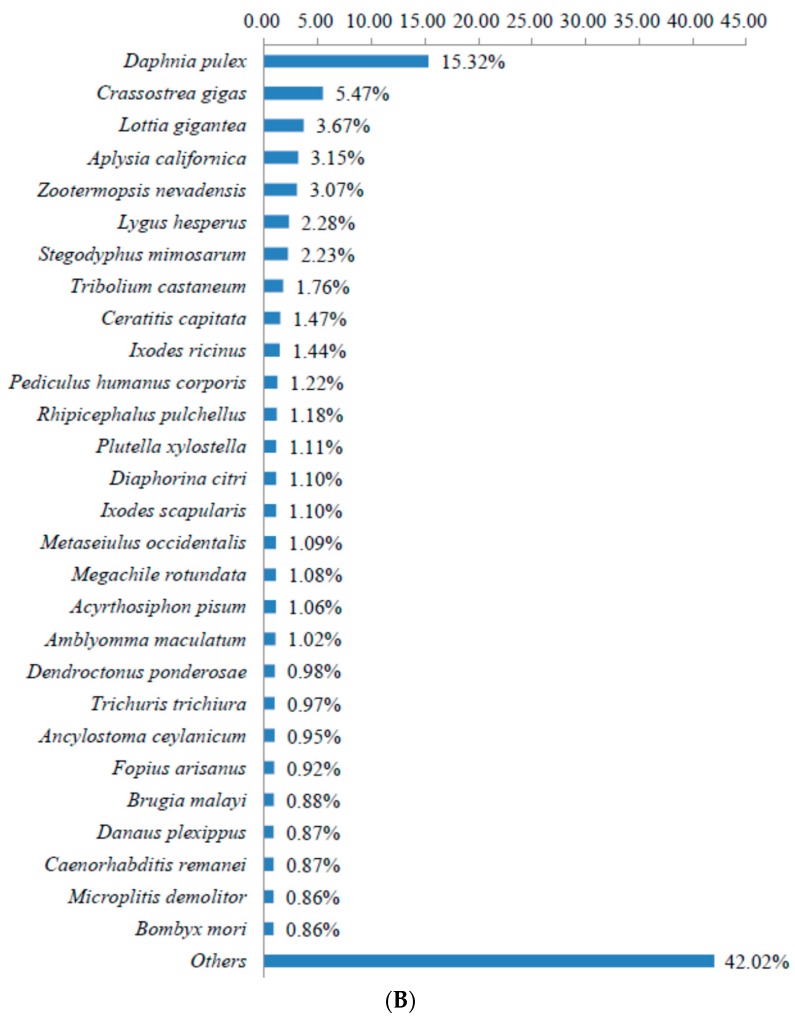
Homology searches of *T. longicaudatus* unigenes against the PANM-DB. (**A**) *E*-value distribution; (**B**) Top-hit species distribution.

The homology search of the unigene sequences of *T. longicaudatus* against PANM-DB using BLASTX was represented of top-hit *E*-values and top-hit species distribution. The *E*-value distribution revealed that 64,493 (73.52%) unigenes showed significant homology to the deposited sequences, with an E-values ranging from 1E−50 to 1E−5 (Figure 3A). For top species distribution, 13,440 (15.32%) unigenes showed similarities with *Daphnia pulex* followed by *Crassostrea gigas* (4794 unigenes; 5.47%), *Lottia gigantea* (3218 unigenes; 3.67%), *Aplysia californica* (2759 unigenes; 3.15%), and others (Figure 3B).

We also examined homology search characteristics such as score, identity and similarity distribution. The score distribution, which represents the quality of the BLAST alignment, showed that 45,158 (51.48%) unigenes had a score <100 (Figure S1A). The identity distribution revealed that 36,329 (41.42%) unigenes showed an identity of 40%–60%, followed by identities of 33.17% and 19.08% for 15%–40% and 60%–80% unigenes, respectively (Figure S1B). According to the similarity distribution analysis, 36,411 (41.51%) unigenes showed a similarity of 60%–80% with homologous sequences in the PANM-DB. Only 18.03% of unigene sequences showed similarity of 80%–100% to sequences in PANM-DB (Figure S1C). The BLASTX annotation hits to homologous protein sequences in PANM-DB increased with increasing unigene length. More than 90% of unigenes with a sequence length >2000 bp showed annotation hits against PANM-DB (Figure S1D).

### 3.3. KOG, GO and KEGG Classifications

**Figure 4:**
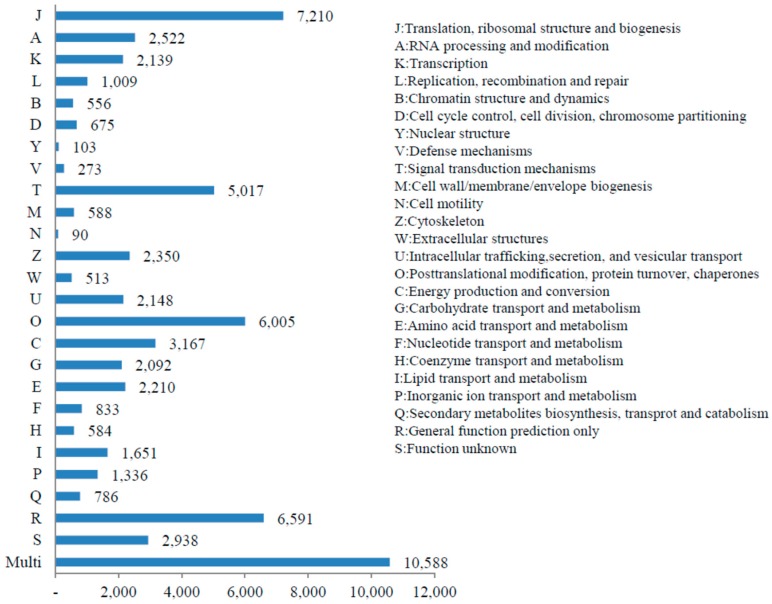
KOG DB based functional analysis of *T. longicaudatus* unigenes.

For a functional classification of the *T. longicaudatus* unigenes, we conducted a BLAST search against the KOG, GO, and KEGG databases. Under the KOG classification, a total of 63,978 unigenes were predicted under 25 functional categories excluding the “multi” category. Within the 25 categories, the unigenes were predominantly distributed to “translation, ribosomal structure and biogenesis (7210 unigenes)”, followed by “general function prediction only” (6591 unigenes), “post-translational modification, protein turnover and chaperones” (6005 unigenes), and “signal transduction mechanisms” (5017 unigenes). The least represented groups included “cell motility” (90 unigenes) and “nuclear structure” (103 unigenes) (Figure 4).

**Figure 5:**
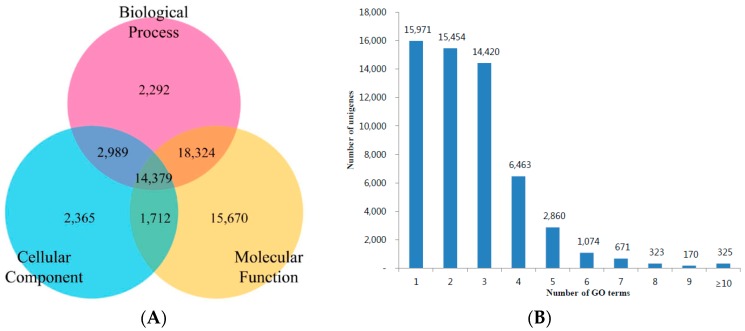
GO term classification for *T. longicaudatus*. (**A**) Predicted functional interpretation of unigenes into represented biological process, cellular component, and molecular function; (**B**) Number of unigene sequences annotated with numbers of GO terms per sequence.

**Figure 6:**
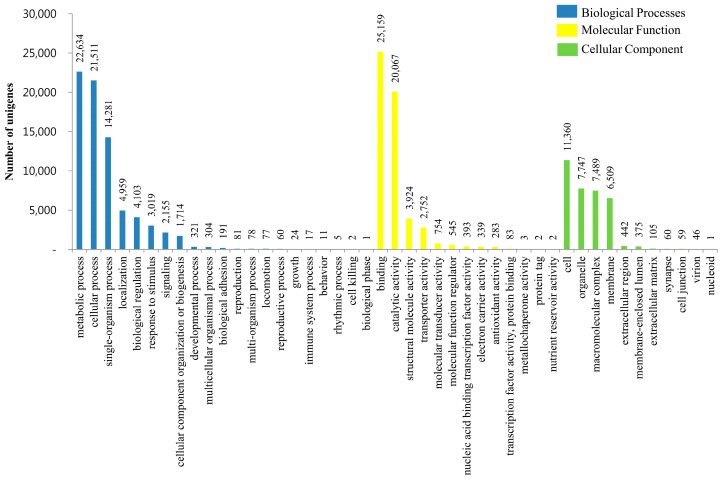
GO annotation of unigenes from *T. longicaudatus* based on biological processes, molecular functions and cellular components.

GO is an international standardization of the gene functional classification system. The GO classification system comprises three large categories: molecular function, biological process and cellular components. Among all the unigenes with GO annotations, we found that 57,731 (27.65% of all unigenes) unigenes matched to GO terms and 14,379 unigenes showed functional attributes shared within the three main categories. The unigenes predominantly shared the biological process and molecular function categories (Figure 5A). Approximately 15,971 (27.7%) unigenes were represented by one GO term; 15,454 (26.8%) unigenes were represented by two GO terms; and 14,420 (25.0%) unigenes were represented by three GO terms of predicted functions (Figure 5B). Additionally, biological processes, molecular functions and cellular components were associated with 75,548, 54,306, and 34,193 unigenes, respectively. In the biological process category, metabolic process (22,634 unigenes), cellular process (21,511 unigenes), and single-organism process (14,281 unigenes) were the most abundant groups, whereas cell killing (2 unigenes) and biological phase (1 unigene) were the least abundant groups. Under the molecular function category, binding (25,159 unigenes) and catalytic activity (20,067 unigenes) were the most abundant groups, while antioxidant activity (283 unigenes) and metallochaperone activity (3 unigenes) were also observed. In cellular component terms, cell (11,360 unigenes), organelle (7747 unigenes), macromolecular complex (7489 unigenes), and membrane (6509 unigenes) were the dominant groups. An account of the suggested function of *T. longicaudatus* unigenes under the GO term categories is shown in Figure 6.

**Figure 7:**
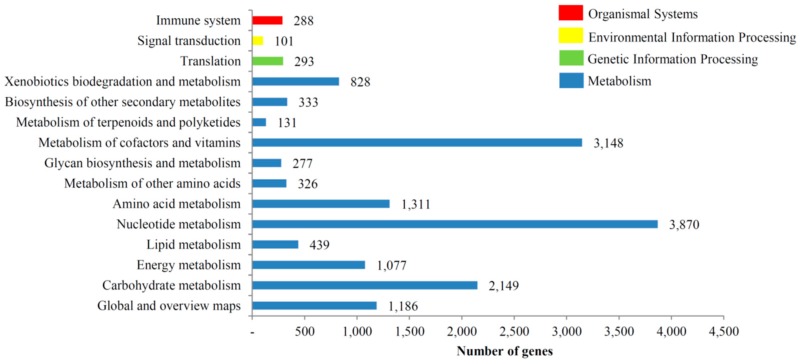
Identified KEGG pathways of assembled unigenes from *T. longicaudatus*.

We classified unigenes into biological pathways by annotating the unigene sequences against the KEGG database. A total of 7247 unigenes were predicted to function in a total of 129 pathways. Predominantly, the unigene sequences were classified into the metabolism pathway group, wherein “nucleotide metabolism”, “metabolism of cofactors and vitamins”, and “carbohydrate metabolism” constituted the major groups (Table S1). A total of 293 unigene sequences were predicted to be classified under translation group, followed by 288 under the immune system and 101 under the signal transduction group. The identified KEGG pathways for *T. longicaudatus* unigenes are presented in Figure 7. Using the InterPro Scan analysis feature in BLAST2GO, we identified the most prominent protein domains predicted for *T. longicaudatus* unigenes. A total of 1252 unigenes showed top-hits to the P-loop-containing nucleoside triphosphate hydrolase (P-loop NTPase) domain. Other top domains identified based on unigene homology included the insulin-like growth factor binding protein, N-terminal domain, zinc finger, C2H2-like domain, heat shock protein 70 family, EGF-like domain, and helicase C-terminal domain (Table S2).

### 3.4. Development and SSR Locus Analysis

To identify SSRs, we scanned 29,547 unigene sequences (75,658,821 bp) of *T. longicaudatus* with lengths >1000 bp. A total of 1595 potential SSR loci were detected including 529 (33.2%), 862 (54%), 144 (9%), 33 (2.1%) and 27 (1.7%), di-, tri, tetra-, penta-, and hexa-nucleotide repeats, respectively (Table 3). The SSR repeats identified were present predominantly in six, five, four, four, and four iterations, respectively, for di-, tri-, tetra-, penta- and hexa-nucleotide repeats (Table S3).

**Figure 8:**
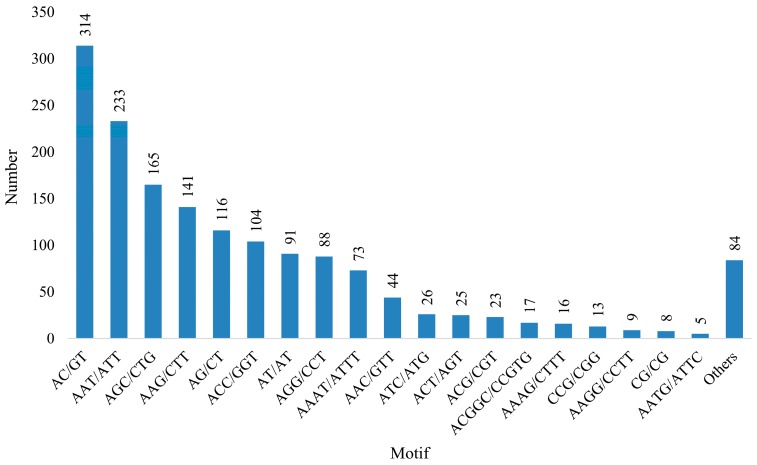
The number of SSRs discovered in the unigenes from *T. longicaudatus* based on motif sequence types.

Among the di-nucleotide repeats, AC/GT (314 unigenes), AG/CT (116 unigenes), and AT/AT (91 unigenes) were the dominant motifs. Within the tri-nucleotide repeats, AAT/ATT (233 unigenes), followed by AGC/CTG (165 unigenes) and AAG/CTT (141 unigenes), were the most repeated motifs (Figure 8). All 1162 SSR-containing unigenes were functionally annotated. In addition, a total of 1387 SSR sites were randomly selected from the SSR-containing sequences to design SSR primers for genotyping. Among the 1387 SSR sites, 1123 were included known functional regions. A list of PCR primers and conditions is shown in Table S4.

## 4. Discussion

In this study, we used high-throughput mRNA-Seq technology to analyze expressed transcripts of the longtail tadpole shrimp *T. longicaudatus*. RNA-Seq platform technology has been used for the rapid characterization of genomic and genetic resources in related non-model species including the Pacific white shrimp (*Litopenaeus vannamei*) [28,42], the Banana shrimp (*Fenneropenaeus merguiensis*) [43], the Brine shrimp (*Artemia franciscana*) [44], and the *Triops newberryi* [1]. Transcriptome studies have also provided advances in establishing putative genes involved in the growth, reproduction and innate immune system pathways in the European shore crab (*Carcinus maenas*) [45], the Mud crab (*Scylla paramamosain*) [46], and the swimming crab (*Portunus trituberculatus*) [47]. These studies have researched the need for genetic data on these species through the screening and exploitation of microsatellites in a cost-efficient and timely manner. In this study, using the Illumina HiSeq 2500 sequencing method and Trinity de novo assembly, 269,822 contigs and 208,813 unigenes were generated. The N_50_ length (1148 bp) and the average length (712.8 bp) of the contigs and unigenes (N_50_ length of 1089 bp and an average length of 700 bp) are greater than in the transcriptomic analysis of other crustacean species such as *L. vannamei* (42,336 unigenes with an N_50_ of 736 bp and an average length of 561 bp) [48], brine shrimp, *A. franciscana* (36,896 contigs with an average length of 746 bp) [44], crayfish, *Cherax quadricarinatus* (36,128 contigs with an N_50_ of 936 bp and an average length of 800 bp) [49], and pandalid shrimp, *Pandalus latirostris* (45,467 contigs with an N_50_ of 493 bp) [50], and are lower than in the transcriptome of *Parhyale hawaiensis* (35,301 contigs with an N_50_ of 1510 bp) [51]. For further we applied the BUSCO, which is reference based software for assessing quality of de novo assembles. Out of 2675 single copy orthologs for arthropods our assembly is 88.56% complete (1708 complete single copy BUSCOs and 661 complete duplicated BUSCOs), while 5.35% of contigs are fragmented (143 fragmented BUSCOs) and 6.09% are missing (163 missing BUSCOs).

We annotated the *T. longicaudatus* unigene sequences against the PANM, Unigene, KOG, GO, and KEGG databases by BLASTX with a cut-off value of 1E−5. Approximately 45.55% of unigenes matched to homologous sequences in the databases, which is less than half of the unigenes present in the *T. longicaudatus* transcriptome could be annotated. Lineage-specific genes are often difficult to annotate because their function is specific to the species [1,52]. We also characterized the homology search using PANM-DB due to the greater degree of annotation of unigene sequences obtained with this database. PANM-DB is preferred over the NCBI nr database due to faster processing of NGS datasets (15 times faster than that of the NCBI nr database) and a higher number of annotation hits [37]. The locally curated PANM-DB was an addition to the Molluscs database, and covers the available sequences of the Protostomia group in a multi-FASTA format [53]. Furthermore, our results showed that more than 90% of unigenes with a sequence length >2000 bp matched with a homologous protein in the databases, which is possible because the protein-coding genes generally give rise to longer full-length transcripts [54]. The BLASTX top-hit species distribution showed putative homology of the annotated unigene sequences across species in the PANM-DB. Most sequences matched the crustacean, *Daphnia pulex* (15.32%), followed by *Crassostrea gigas* (5.47%) and *Lottia gigantea* (3.67%).

Functional annotations of the assembled unigenes using KOG, GO terms, KEGG pathway analysis, as well as an InterPro conserved domain scan, were conducted to obtain a comprehensive description of the properties of these genes and their products in the species. GO classification only suggests that a unigene is related to a predicted function, as all GO terms are not of equal validity [55]. Most of the evidence codes are based on electronic annotations and are not manually created. The computational source of evidence constitutes more than 95% of the total GO annotation results in non-model species [56,57]. KEGG pathway analysis suggests the classification of unigenes into regulatory biological pathways that include metabolism, genetic information processing, environmental information processing, and organismal systems. The *T. longicaudatus* unigenes were mapped to 129 reference canonical pathways, among which distribution to the metabolism pathways was predominant. In the transcriptome analysis of *Litopenaeus vannamei*, a total of 9621 unigenes were mapped to 317 pathways, wherein the most enriched sequences were assigned to metabolic pathways, followed by the biosynthesis of secondary metabolites and spliceosome and RNA transport [58]. In the mud crab (*Scylla paramamosain*) transcriptome using 454 sequencing, 4878 unigenes were classified into 281 KEGG pathways, and the identified genes were found to be involved in growth, development, and disease resistance pathways [46]. Among the top-hit InterPro domain obtained in the present analysis, P-loop NTPases were predominant. These represent a large protein family that is involved in a variety of cellular functions, such as signal transduction, translation, protein transport and localization, signal-sequence recognition, chromosome partitioning, and membrane transport [59]. The C_2_H_2_ type zinc finger domains are widely found in DNA binding motifs in eukaryotic transcription factors [60].

Polymorphic microsatellite markers such as SSRs have been utilized for a variety of genetic and breeding studies [61]. NGS technologies can be used to develop abundant SSR or SNP markers with high efficiency and accuracy [62]. In this study, we screened 1595 SSRs of 2–6 bp in length from unigene sequences >1000 bp in length. The tri-nucleotide repeats were predominant, followed by di- and tetra-nucleotide repeats. The tri-nucleotide SSR motifs have been consistently found as the predominant markers in the transcriptome sequences of many monocotyledonous plants [63,64]; however, in animals, the di-nucleotide repeats are predominant [65]. One nucleotide repeat motifs were detected but were not considered as these may be the result of single nucleotide stretch errors generated by sequencing [66,67]. These SSR loci provide an abundant marker resource for studying the genetic variation, population, and conservation genomics of species. In a previous study that constructed a genetic linkage map of *L*. *vannamei* using AFLP and SSR markers, 25 SSR markers were found to be informative in mapping a population of *L. vannamei* and are available for map construction [68]. The abundance of AC/GT motifs found in the present study is consistent with the SSR motif study in the mud crab, *Scylla paramamosain* [46]. The tri-nucleotide motifs AGC/CTG and ACC/GGT found in this study were also the preferred motifs in the SSRs isolated from the transcriptome of the Red Swamp Crayfish *Procambarus clarkii* [69]. A total of 1387 potential SSR markers identified in this study will provide important research advances for genetic studies including the assessment of genetic diversity, the development of genetic maps, comparative genomics, and marker-assisted selection breeding. The primer pairs designed for polymorphism identification would add towards genotyping of the species diversity and exploitation of the economic potential of the species.

## 5. Conclusions

This is the first report of high-throughput transcriptome analysis of *T. longicaudatus*. In total, 95,105 unigenes were annotated for putative functions using BLASTX with a cut-off of 1E−5. A total of 57,731 unigenes were assigned to GO terms, and 7247 unigenes were mapped to 129 KEGG pathways. Furthermore, 1595 SSRs were detected from the unigenes with 1387 potential SSR markers. A total of 1387 potential SSR markers identified in this study will provide important research advances for genetic studies including the assessment of genetic diversity, the development of genetic maps, comparative genomics, and marker assisted selection breeding.

## Figures and Tables

**Table 1:** Summary statistics from Illumina sequencing of the *T. longicaudatus*.

Total Number of Raw Reads	
Number of sequences	323,319,608
Number of bases	40,738,270,608
Total number of clean reads	
Number of sequences	318,610,596
Number of bases	39,745,513,470
Mean length of contig (bp)	124.7
N_50_ length of contig (bp)	126
GC % of contig	48.39
High-quality reads (%)	98.54 (sequences), 97.56 (bases)
Contig information	
Total number of contig	269,822
Number of bases	192,327,026
Mean length of contig (bp)	712.8
N_50_ length of contig (bp)	1148
GC % of contig	46.82
Largest contig (bp)	40,450
No. of large contigs (≥500 bp)	89,407
Unigene information	
Total number of unigenes	208,813
Number of bases	146,173,633
Mean length of unigene (bp)	700.0
N_50_ length of unigene (bp)	1089
GC % of unigene	46.97
Length ranges (bp)	224–40,450

**Table 2:** Annotation of *T. longicaudatus* assembled unigene sequences against public databases.

Databases	All	≤300 bp	300–1000 bp	≥1000 bp
PANM-DB	87,719	20,029	43,958	23,732
UNIGENE	26,845	6231	12,885	7729
KOG	63,978	12,955	30,892	20,131
GO	57,731	12,915	28,153	16,663
KEGG	7247	1735	3400	2112
ALL	95,105	22,935	48,081	24,089

The number of unigenes hits using BLASTX search (*E*-value < 1E−5).

**Table 3:** SSRs identified from the unigene sequences of *T. longicaudatus*.

SSR parameters	Number
Total number of sequences examined	29,547
Total size of examined sequences (bp)	75,658,821
Total number of identified SSRs	1595
Di-nucleotide	529
Tri-nucleotide	862
Tetra-nucleotide	144
Penta-nucleotide	33
Hexa-nucleotide	27
Number of SSR containing sequences	1432
Number of sequences containing more than 1 SSR	140
Number of SSRs present in compound formation	74

## Supplementary Materials

The following are available online at www.mdpi.com/2073-4425/7/12/114/s1. Figure S1: Summary of homology search of assembled unigenes of *T. longicaudatus* against PANM-DB. (A) score distribution, (B) identity distribution, (C) similarity distribution, (D) distribution of hit and non-hit sequences as compared with the length of unigenes; Table S1: KEGG mappings for *T. longicaudatus* unigenes; Table S2: List of top-hit InterPro domains in *T. longicaudatus*, Table S3: Summary of SSR types in the *T. longicaudatus* transcriptome, Table S4: Sequences of 1,387 primer pairs for SSR markers.

## References

[1] Horn R.L., Ramaraj T., Devitt N.P., Schilkey F.D., Cowley D.E. (2016). De novo assembly of a tadpole shrimp (*Triops newberryi*) transcriptome and preliminary differential gene expression analysis. Mol. Ecol. Resour..

[2] Suno-Uchi N., Sasaki F., Chiba S., Kawata M. (1997). Morphological stasis and phylogenetic relationships in Tadpole shrimps, *Triops* (Crustacea: Notostraca). Biol. J. Linn. Soc..

[3] Wooten D. (2010). *Triops* *longicaudatus*. Zooplankton of the Great Lakes.

[4] Fry L.L., Mulla M.S., Adams C.W. (1994). Field introductions and establishment of the Tadpole shrimp, *Triops longicaudatus* (Notostraca: Triopsidae), a biological control agent of mosquitoes. Biol. Control.

[5] Becker N., Petric D., Zgomba M., Boase C., Madon M., Dahl C., Kaiser A. (2010). Mosquitoes and Their Control.

[6] Yonekura M. Weeding efficacy of tadpole shrimp (*Triops* spp.) in transplanted rice fields. In Proceedings of the 7th Asian Pacific Weed Science Society Conference.

[7] Korn M., Marrone F., Perez-Bote J.L., Machado M., Cristo M., Da Fonseca L.C., Hundsdoerfer A.K. (2006). Sister species within the *Triops cancriformis* lineage (Crustacea, Notostraca). Zool. Scr..

[8] Macdonald K.S., Sallenave R., Cowley D.E. (2011). Morphologic and Genetic variation in *Triops* (Branchiopoda: Notostraca) from ephemeral waters of the Northern Chihuahuan Desert of North America. J. Crustacean Biol..

[9] Horn R.L., Cowley D.E. (2014). Evolutionary relationships within *Triops* (Branchiopoda: Notostraca) using complete mitochondrial genomes. J. Crustacean Biol..

[10] Linder F. (1952). Contributions to the morphology and taxonomy of the Branchiopoda Notostraca, with special reference to the North American species. Proc. U. S. Natl. Mus..

[11] Longhurst A.R. (1955). A review of the Notostraca. Bulletin of the British Museum (Natural History). Zoology.

[12] Akita M. (1976). Classification of Japanese tadpole shrimps. Zool. Mag..

[13] Yoon S.M., Kim W., Kim H.S. (1992). Re-description of *Triops longicaudatus* (LeConte, 1846) (Notostraca, Triopsidae) from Korea. Korean J. Syst. Zool..

[14] Sassaman C., Simovich M., Fugate M. (1997). Reproductive isolation and genetic differentiation in North American species of *Triops* (Crustacea: Branchiopoda: Notostraca). Hydrobiologia.

[15] Kwon S.J., Jun Y.C., Park J.H., Won D.H., Seo E.W., Lee J.E. (2010). Distribution and habitat characteristics of tadpole Shrimp. Korean J. Limnol..

[16] Aloufi A.B.A., Obuid-Allah A.H. (2014). New records and Redescription of the notostracan Tadpole shrimp, *Triops longicaudatus* (LeConte, 1846) from temporary water bodies in North West region (Tabuk and Madinah) in Saudi Arabia. Int. J. Adv. Res..

[17] Kwon S.J., Kwon H.J., Jun Y.C., Lee J.E., Won D.H. (2009). Effect of temperature on hatching rate of *Triops longicaudatus* (Triopsidae, Notostraca). Korean J. Limnol..

[18] Ryu J.S., Hwang U.W. (2010). Complete mitochondrial genome of the longtail tadpole shrimp *Triops longicaudatus* (Crustacea, Branchiopoda, Notostraca). Mitochondrial DNA.

[19] Wu P., Qi D., Chen L., Zhang H., Zhang X., Qin J.G., Hu S. (2009). Gene discovery from an ovary cDNA library of oriental river prawn *Macrobrachium nipponense* by ESTs annotation. Comp. Biochem. Physiol. Part D Genom. Proteom..

[20] Metzker M.L. (2010). Sequencing technologies—The next generation. Nat. Rev. Genet..

[21] Qi X., Zhang L., Han Y., Ren X., Huang J., Chen H. (2015). De novo transcriptome sequencing and analysis of *Coccinella septempunctata L.* in non-diapause, diapause and diapause-terminated states to identify diapause-associated genes. BMC Genom..

[22] Jin H., Dong D., Yang Q., Zhu D. (2016). Salt-responsive transcriptome profiling of *Suaeda glauca* via RNA Sequencing. PLoS ONE.

[23] Patnaik B.B., Hwang H.-J., Kang S.W., Park S.Y., Wang T.H., Park E.B., Chung J.M., Song D.K., Kim C., Kim S. (2015). Transcriptome Characterization for non-model endangered Lycaenids, *Protantigius superans* and *Spindasis takanosis*, using Illumina HiSeq 2500 Sequencing. Int. J. Mol. Sci..

[24] Morozova O., Marra M.A. (2008). Applications of Next-Generation sequencing technologies in functional genomics. Genomics.

[25] Novaes E., Drost D.R., Farmerie W.G., Pappas G.J., Grattapaglia D., Sederoff R.R., Kirst M. (2008). High-throughput gene and SNP discovery in *Eucalyptus grandis*, an uncharacterized genome. BMC Genom..

[26] Zimmer C.T., Malwald F., Schorn C., Bass C., Ott M.C., Nauen R. (2014). A de novo transcriptome of European pollen beetle populations and its analysis, with special reference to insecticide action and resistance. Insect Mol. Biol..

[27] Fullwood M.J., Wei C.L., Liu E.T., Ruan Y. (2009). Next-generation DNA sequencing of paired-end tags (PET) for transcriptome and genome analyses. Genome Res..

[28] Li D.J., Deng Z., Qin B., Liu X.H., Men Z.H. (2012). De novo assembly and characterization of bark transcriptome using Illumina sequencing and development of EST–SSR markers in rubber tree (*Hevea brasiliensis* Muell. Arg.). BMC Genom..

[29] Sadamoto H., Takahashi H., Okada T., Kenmoku H., Toyota M., Asakawa Y. (2012). De novo Sequencing and transcriptome analysis of the Central Nervous System of Mollusc *Lymnaea stagnalis* by Deep RNA Sequencing. PLoS ONE.

[30] Che R., Sun Y., Wang R., Xu T. (2014). Transcriptomic analysis of endangered Chinese salamander: Identification of immune, sex and reproduction-related genes and genetic markers. PLoS ONE.

[31] Joshi N.A., Fass J.N. Sickle: A Sliding-Window, Adaptive, Quality-Based Trimming Tool for FastQ Files (Version 1.33) [Software]. https://github.com/najoshi/sickle.

[32] Martin M. (2011). Cutadapt removes adapter sequences from high-throughput sequencing reads. EMBnet.journal.

[33] Haas B.J., Papanicolaou A., Yassour M., Grabherr M., Blood P.D., Bowden J., Couger M.B., Eccles D., Li B., Lieber M. (2013). De novo transcript sequence reconstruction from RNA-seq using the Trinity platform for reference generation and analysis. Nat. Protoc..

[34] Surget-Groba Y., Montoya-Burgos J.I. (2010). Optimization of de novo transcriptome assembly from next-generation sequencing data. Genome Res..

[35] Hao D.C., Ge G.B., Xiao P.G., Zhang Y.Y., Yang L. (2011). The first insight into the Taxus genome via fosmid library construction and end sequencing. Mol. Genet. Genom..

[36] Simão F.A., Waterhouse R.M., Ioannidis P., Kriventseva E.V., Zdobnov E.M. (2015). BUSCO: Assessing genome assembly and annotation completeness with single-copy orthologs. Bioinformatics.

[37] Kang S.W., Patnaik B.B., Hwang H.J., Park S.Y., Lee J.S., Han Y.S., Lee Y.S. (2015). PANM DB (Protosome DB) for the annotation of NGS data of mollusks. Korean J. Malacol..

[38] Camacho C., Coulouris G., Avagyan V., Ma N., Papadapoulous J., Bealer K., Madden T.L. (2009). BLAST+: Architecture and applications. BMC Bioinform..

[39] Conesa A., Gotz S., Garcia-Gomez J.M., Terol J., Talon M., Robles M. (2005). Blast2GO: A universal tool for annotation, visualization and analysis in functional genomics research. Bioinformatics.

[40] Ye J., Fang L., Zheng H., Zhang Y., Chen J., Zhang Z., Wang J., Li S., Li R., Bolund L. (2006). WEGO: A web tool for plotting GO annotations. Nucleic Acids Res..

[41] Kanehisa M., Goto S., Kawashima S., Okuno Y., Hattori M. (2004). The KEGG resource for deciphering the genome. Nucleic Acids Res..

[42] Chen K., Li E., Xu Z., Li T., Xu C., Qin J.G., Chen L. (2015). Comparative Transcriptome analysis in the hepatopancreas tissue of Pacific White Shrimp *Litopenaeus vannamei* fed different lipid sources at low salinity. PLoS ONE.

[43] Powell D., Knibb W., Remilton C., Elizur A. (2015). De-novo transcriptome analysis of the banana shrimp (*Renneropenaeus merguiensis*) and identification of genes associated with reproduction and development. Mar. Genom..

[44] Valenzuela-Miranda D., Gallardo-Escarate C., Valenzuela-Munoz V., Farlora R., Gajardo G. (2014). Sex-dependent transcriptome analysis and single nucleotide polymorphism (SNP) discovery in the brine shrimp *Artemia franciscana*. Mar. Genom..

[45] Verbruggen B., Bickley L.K., Santos E.M., Tyler C.R., Stentiford G.D., Bateman K.S., van Aerle R. (2015). De novo assembly of the *Carcinus maenus* transcriptome and characterization of innate immune system pathways. BMC Genom..

[46] Ma H., Ma C., Li S., Jiang W., Li X., Liu Y., Ma L. (2014). Transcriptome analysis of the Mud Crab (*Scylla paramamosain*) by 454 deep sequencing: Assembly, Annotation and Marker Discovery. PLoS ONE.

[47] Lv J., Liu P., Gao B., Wang Y., Wang Z., Chen P., Li J. (2014). Transcriptome analysis of the *Portunus trituberculatus*: De novo assembly, growth-related gene identification and marker discovery. PLoS ONE.

[48] Guo H., Ye C.X., Wang A.L., Xian J.A., Liao S.A., Miao Y.T., Zhang S.P. (2013). Transcriptome analysis of the Pacific white shrimp *Litopenaeus vannamei* exposed to nitrite by RNA-seq. Fish Shellfish Immunol..

[49] Ali M.Y., Pavasovic A., Mather P.B., Prentis P.J. (2015). Transcriptome analysis and characterization of gill-expressed carbonic anhydrase and other key osmoregulatory genes in freshwater crayfish *Cherax quadricarinatus*. Data Brief.

[50] Ryouka K.-M., Wada K., Azuma N., Chiba S. (2011). Expression profiling without genome sequence information in a non-model species, Pandalid shrimp (*Pandalus latirostris*), by next-generation sequencing. PLoS ONE.

[51] Zeng V., Villaneuva K.E., Ewen-Campen B.S., Alwes F., Browne W.E., Extavour C.G. (2011). De novo assembly and characterization of a maternal and developmental transcriptome for the emerging model crustacean *Parhyale hawaiensis*. BMC Genom..

[52] Asselman J., Pfrender M.E., Lopez J.A., De Coninck D.I., Janssen C.R., Shaw J.R., De Schamophelaere K.A. (2015). Conserved transcriptional responses to cyanobacterial stressors are mediated by alternate regulation of paralogous genes in *Daphnia*. Mol. Ecol..

[53] Kang S.W., Hwang H.J., Park S.Y., Wang T.H., Park E.B., Lee T.H., Hwang U.W., Lee J.S., Park H.S., Han Y.S. (2014). Mollusks sequence database: Version II. Korean J. Malacol..

[54] Hwang H.-J., Patnaik B.B., Kang S.W., Park S.Y., Wang T.H., Park E.B., Chung J.M., Song D.K., Patnaik H.H., Kim C. (2016). RNA sequencing, de novo *assembly*, and functional annotation of an endangered Nymphalid butterfly, *Fabriciana nerippe* Felder, 1862. Entomol. Res..

[55] Rhee S.Y., Wood V., Dolinski K., Draghici S. (2008). Use and misuse of the gene ontology annotations. Nat. Rev. Genet..

[56] Patnaik B.B., Wang T.H., Kang S.W., Hwang H.-J., Park S.Y., Park E.B., Chung J.M., Song D.K., Kim C., Kim S. (2016). Sequencing, de novo assembly, and annotation of the transcriptome of the endangered freshwater pearl bivalve, *Cristaria plicata*, provides novel insights into functional genes and marker discovery. PLoS ONE.

[57] Park S.Y., Patnaik B.B., Kang S.W., Hwang H.-J., Chung J.M., Song D.K., Sang M.K., Patnaik H.H., Lee J.B., Noh M.Y. (2016). Transcriptome analysis of the endangered neritid species *Clithon retropictus*: De novo Assembly, Functional Annotation, and Marker Discovery. Genes.

[58] Chen K., Li E., Li T., Xu C., Wang X., Lin H., Qin J.G., Chen L. (2015). Transcriptome and pathway analysis of the Hepatopancreas in the Pacific White Shrimp *Litopenaeus vannamei* under Chronic Low-Salinity stress. PLoS ONE.

[59] Pathak E., Atri N., Mishra R. (2013). Role of highly central residues of P-loop and it’s flanking region in preserving the archetypal conformation of Walker A motif of diverse P-loop NTPases. Bioinformation.

[60] Razin S.V., Borunova V.V., Maksimenko O.G., Kantidze O.L. (2012). Cys2His2 Zinc Finger Protein Family: Classification, Functions, and Major Members. Biochemistry (Moscow).

[61] Luo H., Xiao S., Ye H., Zhang Z., Lv C., Zheng S., Wang Z., Wang X. (2016). Identification of immune related genes and development of SSR/SNP markers from the spleen transcriptome of Schizothorax prenanti. PLoS ONE.

[62] Liang M., Yang X., Li H., Su S., Yi H., Chai L., Deng X. (2015). De novo transcriptome assembly of pummelo and molecular marker development. PLoS ONE.

[63] La Rota M., Kantety R.V., Yu J.K., Sorrells M.E. (2005). Nonrandom distribution and frequencies of genomic and EST-derived microsatellite markers in rice, wheat, and barley. BMC Genom..

[64] Annadurai R.S., Neethiraj R., Jayakumar V., Damodaran A.C., Rao S.N., Katta M.A.V.S.K., Gopinathan S., Sarma S.P., Senthilkumar V., Niranjan V. (2013). De novo transcriptome assembly (NGS) of *Curcuma longa* L. rhizome reveals novel transcripts related to anticancer and antimalarial terpenoids. PLoS ONE.

[65] Chen M., Tan Z., Zeng G., Peng J. (2010). Comprehensive analysis of simple sequence repeats in pre-miRNAs. Mol. Biol. Evol..

[66] Miller A.D., Good R.T., Coleman R.A., Lancaster M.L., Weeks A.R. (2013). Microsatellite loci and the complete mitochondrial DNA sequence characterized through next generation sequencing and de novo assembly for the critically endangered orange-bellied parrot, *Neophema chrysogaster*. Mol. Biol. Rep..

[67] Zhang S.H., Luo H., Du H., Wang D.Q., Wei Q.W. (2013). Isolation and characterization of twenty-six microsatellite loci for the tetraploid fish Dabry’s sturgeon (*Acipenser dabryanus*). Conserv. Genet. Res..

[68] Andriantahina F., Liu X., Huang H. (2013). Genetic map construction and quantitative trait locus (QTL) detection of growth-related traits in *Litopenaeus vannamei* for selective breeding applications. PLoS ONE.

[69] Shen D., Bo W., Xu F., Wu R. (2014). Genetic diversity and population structure of the Tibetan poplar (*Populus szechuanica* var. *tibetica*) along an altitude gradient. BMC Genet..

