# Supplementary material for: Transcriptome Analysis of the Tadpole Shrimp (Triops longicaudatus) by Illumina Paired-End Sequencing: Assembly, Annotation, and Marker Discovery
Source: Genes (Basel). 2016 Dec 2;7(12):114. doi: 10.3390/genes7120114 (PMC5192490; doi:10.3390/genes7120114)
Supplement: Supplementary file 1 [file genes-07-00114-s001.zip › genes-152720-supplementary-final/genes-152720-suppl-done.docx]

Transcriptome Analysis of the Tadpole Shrimp (*Triops longicaudatus*) by Illumina Paired-End Sequencing: Assembly, Annotation,
and Marker Discovery

Jiyeon Seong, Se Won Kang, Bharat Bhusan Patnaik, So Young Park, Hee Ju Hwang,
Jong Min Chung, Dae Kwon Song, Mi Young Noh, Seung-Hwan Park, Gwang Joo Jeon,
Hong Sik Kong, Soonok Kim, Ui Wook Hwang, Hong Seog Park, Yeon Soo Han
and Yong Seok Lee

| **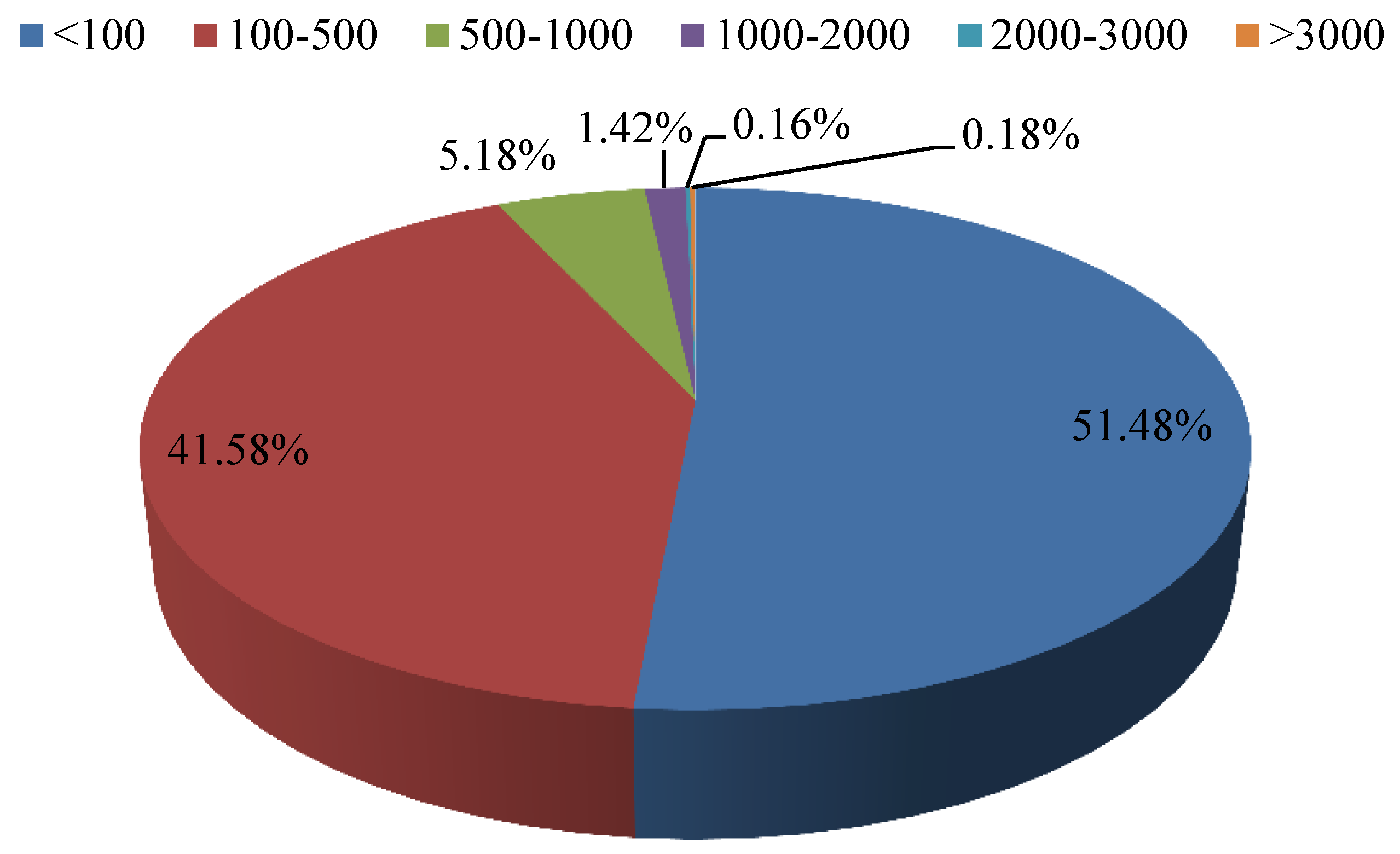** | **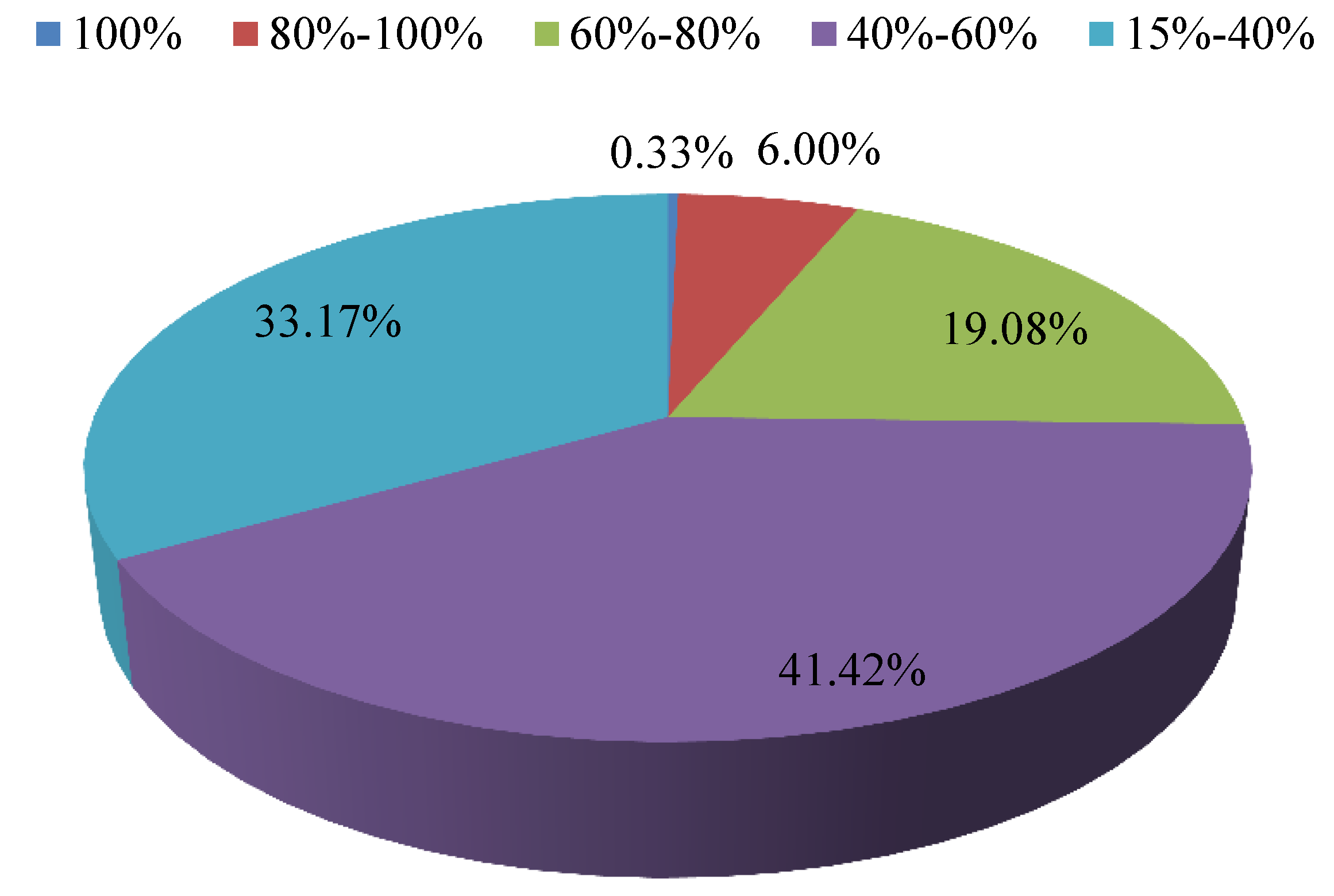** |
| --- | --- |
| (**A**) | (**B**) |
| 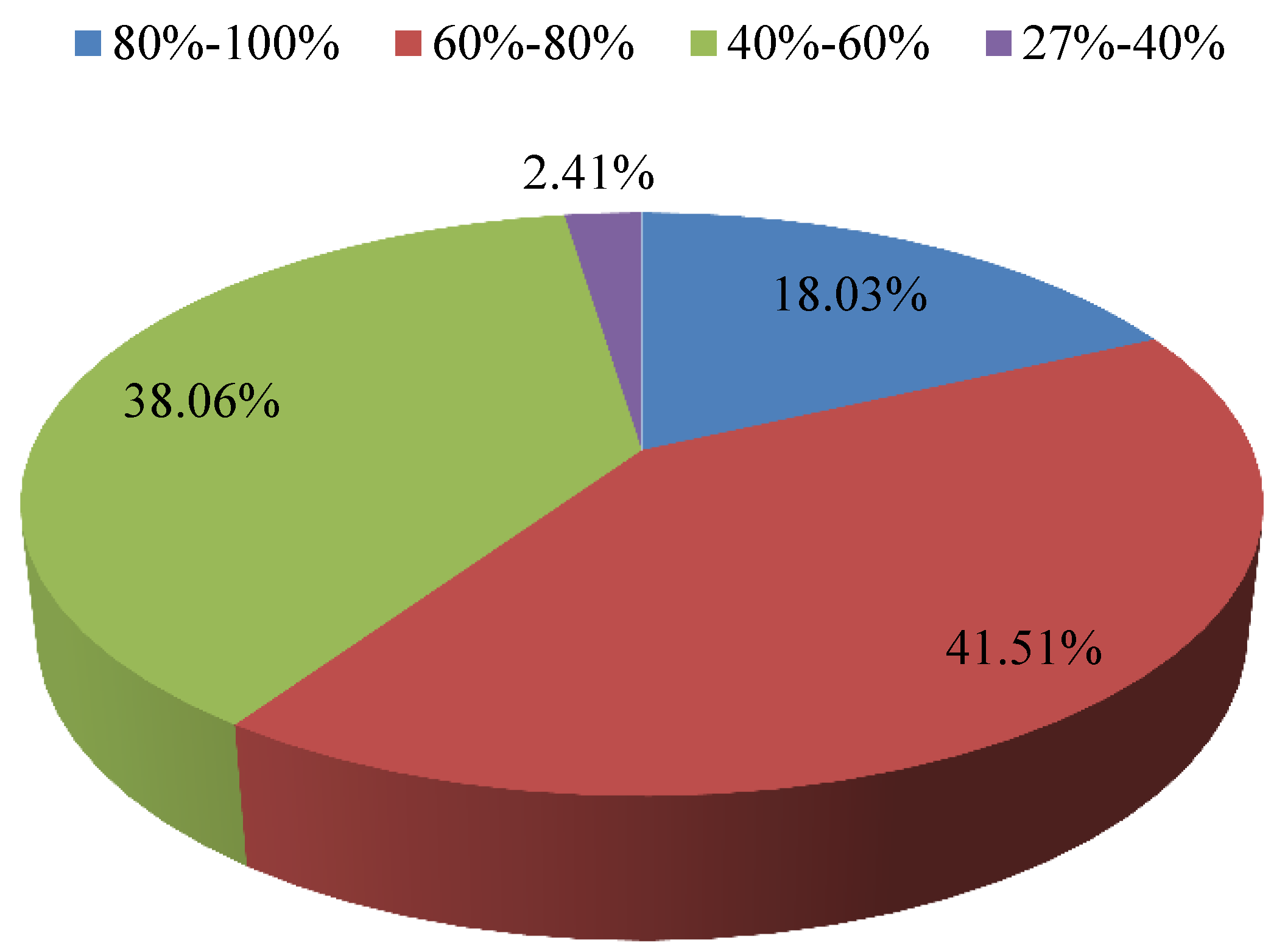 | 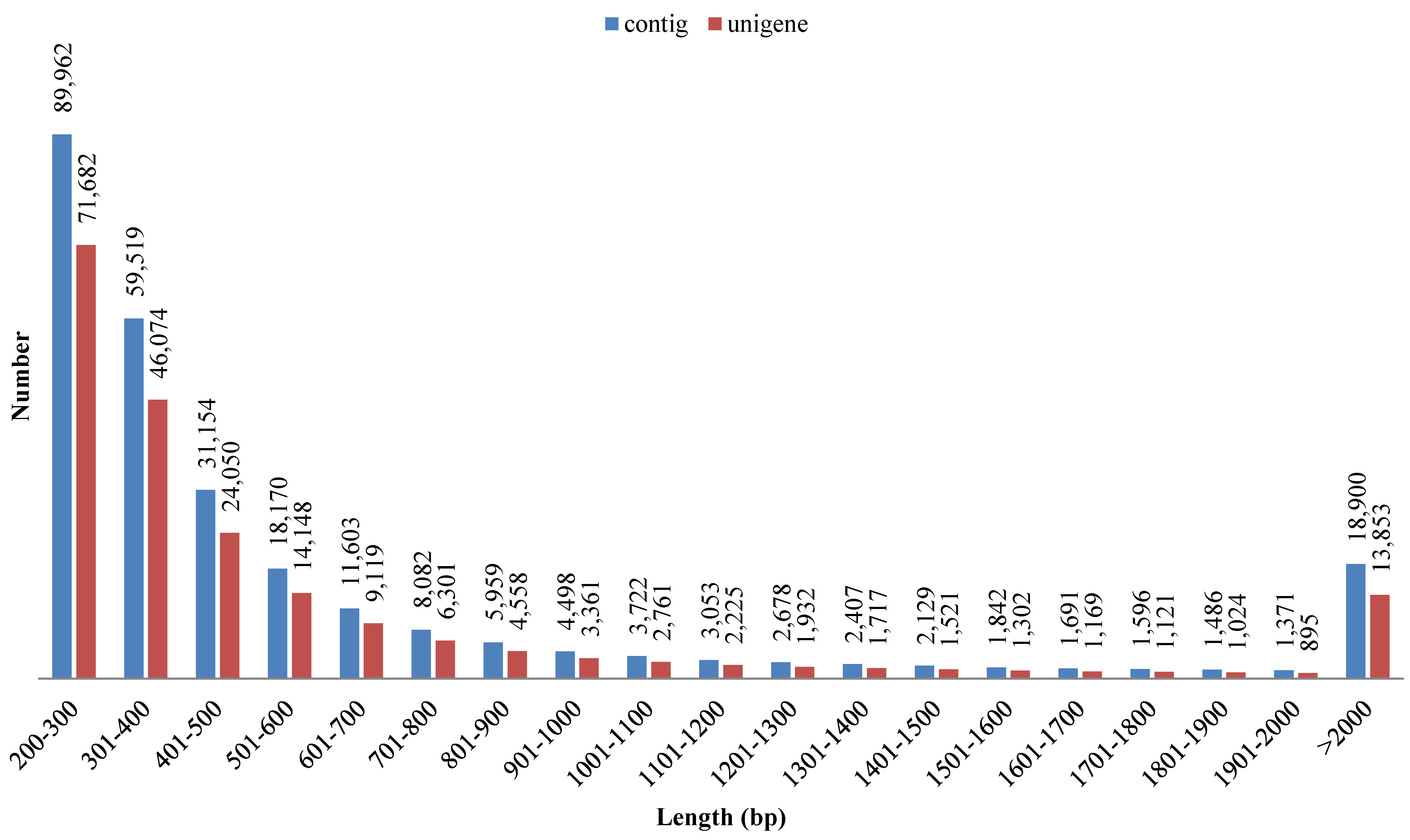 |
| (**C**) | (**D**) |

**Figure S1.** Summary of homology search of assembled unigenes of *T. longicaudatus* against
PANM-DB. (**A**) score distribution; (**B**) identity distribution; (**C**) similarity distribution; (**D**) distribution of hit and non-hit sequences as compared with the length of unigenes.

**Table S1.** KEGG mappings for *T. longicaudatus* unigenes.

|  | | **Seqs in Pathway** | **Enzyme in Pathway** | **Seqs of Enzyme** |
| --- | --- | --- | --- | --- |
| **Metabolism** | Global and overview maps | 1186 | 132 | 1251 |
|  | Carbohydrate metabolism | 2149 | 208 | 2297 |
|  | Energy metabolism | 1077 | 70 | 1158 |
|  | Lipid metabolism | 439 | 65 | 454 |
|  | Nucleotide metabolism | 3870 | 71 | 5055 |
|  | Amino acid metabolism | 1311 | 187 | 1374 |
|  | Metabolism of other amino acids | 326 | 40 | 328 |
|  | Glycan biosynthesis and metabolism | 277 | 33 | 277 |
|  | Metabolism of cofactors and vitamins | 3148 | 81 | 3153 |
|  | Metabolism of terpenoids and polyketides | 131 | 24 | 132 |
|  | Biosynthesis of other secondary metabolites | 333 | 39 | 394 |
|  | Xenobiotics biodegradation and metabolism | 828 | 40 | 889 |
|  | Translation | 293 | 21 | 295 |
| **Environmental Information Processing** | Signal transduction | 101 | 10 | 102 |
| **Organismal Systems** | Immune system | 288 | 2 | 288 |
| **Sum** |  | **15,757** | **1023** | **17,447** |

**Table S2.** List of top-hit InterPro domains in *Triops longicaudatus*. (See Excel Table S2)

**Table S3.** Summary of SSR types in the *T. longicaudatus* transcriptome.

| **Repeats** | **4** | **5** | **6** | **7** | **8** | **9** | **10** | **11** | **12** | **13** | **14** | **15** | **16** | **17** | **18** | **19** | **20** | **≥21** | **Total** |
| --- | --- | --- | --- | --- | --- | --- | --- | --- | --- | --- | --- | --- | --- | --- | --- | --- | --- | --- | --- |
| Di | 0 | 0 | 240 | 136 | 41 | 46 | 17 | 19 | 8 | 5 | 5 | 1 | 0 | 3 | 1 | 0 | 0 | 7 | 529 |
| Tri | 0 | 521 | 216 | 56 | 23 | 10 | 7 | 0 | 2 | 1 | 3 | 0 | 1 | 4 | 5 | 1 | 3 | 9 | 862 |
| Tetra | 125 | 17 | 2 | 0 | 0 | 0 | 0 | 0 | 0 | 0 | 0 | 0 | 0 | 0 | 0 | 0 | 0 | 0 | 144 |
| Penta | 31 | 2 | 0 | 0 | 0 | 0 | 0 | 0 | 0 | 0 | 0 | 0 | 0 | 0 | 0 | 0 | 0 | 0 | 33 |
| Hexa | 25 | 1 | 0 | 1 | 0 | 0 | 0 | 0 | 0 | 0 | 0 | 0 | 0 | 0 | 0 | 0 | 0 | 0 | 27 |
| Total | 181 | 541 | 458 | 193 | 64 | 56 | 24 | 19 | 10 | 6 | 8 | 1 | 1 | 7 | 6 | 1 | 3 | 16 | 1595 |

**Table S4.** Sequences of 1387 primer pairs for SSR markers. (See Excel Table S4)
